# Experimental manipulation of infant temperament affects amygdala functional connectivity

**DOI:** 10.3758/s13415-017-0518-8

**Published:** 2017-06-05

**Authors:** Madelon M. E. Riem, Marinus H. Van Ijzendoorn, Christine E. Parsons, Katherine S. Young, Pietro De Carli, Morten L. Kringelbach, Marian J. Bakermans-Kranenburg

**Affiliations:** 1 Department of Medical and Clinical Psychology, Tilburg University, Tilburg, The Netherlands; 2 Centre for Child and Family Studies, Leiden University, Leiden, The Netherlands; 3 Leiden Institute for Brain and Cognition, Leiden University, Leiden, The Netherlands; 4 Interacting Minds Center, Department of Clinical Medicine, Aarhus University, Aarhus, Denmark; 5 Department of Psychiatry, University of Oxford, Oxford, UK; 6 Department of Psychology, University of California, Los Angeles, CA USA; 7 University of Milano-Bicocca, Milano, Italy

**Keywords:** Amygdala, fMRI, Functional connectivity, Infant temperament, Reward

## Abstract

In this functional magnetic resonance imaging (fMRI) study we examined neural processing of infant faces associated with a happy or a sad temperament in nulliparous women. We experimentally manipulated adult perception of infant temperament in a probabilistic learning task. In this task, participants learned about an infant's temperament through repeated pairing of the infant face with positive or negative facial expressions and vocalizations. At the end of the task, participants were able to differentiate between “mostly sad” infants who cried often and “mostly happy” infants who laughed often. Afterwards, brain responses to neutral faces of infants with a happy or a sad temperament were measured with fMRI and compared to brain responses to neutral infants with no temperament association. Our findings show that a brief experimental manipulation of temperament can change brain responses to infant signals. We found increased amygdala connectivity with frontal regions and the visual cortex, including the occipital fusiform gyrus, during the perception of infants with a happy temperament. In addition, amygdala connectivity was positively related to the post-manipulation ratings of infant temperament, indicating that amygdala connectivity is involved in the encoding of the rewarding value of an infant with a happy temperament.

## Introduction

While all infants cry, they differ from each other in how frequently and intensely they cry. Infants with a temperament characterized by negative emotionality cry more often and tend to react to stressors with a high degree of emotionality, including anger, irritability, fear, or sadness (Rothbart, Ahadi, & Hershey, 1994). Research has shown that infant temperament influences the way parents respond to their infant. For example, irritable or demanding children may elicit feelings of irritation in parents and withdrawal of contact (Putnam, Samson, & Rothbart, 2002). In a meta-analysis, Paulussen-Hoogeboom, Stams, Hermanns, and Peetsma (2007) showed that negative emotionality is associated with insensitive parenting in families with low socioeconomic status. This can in turn lead to later problems with a child’s emotional functioning (Paulussen-Hoogeboom, Stams, Hermanns, Peetsma, & Van Den Wittenboer, 2008). However, previous studies examining the relation between infant temperament and caregiver perception and behavior are correlational and parent measures of infant temperament may be influenced by parental negative mood (Youngstrom, Izard, & Ackerman, 1999). In natural environments, it is not possible to experimentally manipulate infant temperament to examine its influence on parenting. Here we examine the effects of computerized manipulation of infant temperamental features on adults’ neural processing of infant faces as assessed with functional magnetic resonance imaging (fMRI).

Neuroimaging studies may provide more insight into the perception of infant signals and point to a role of the amygdala (for reviews see Rilling, 2013; Swain et al., 2014), a brain region involved in the processing of arousal, threat, or fear, and the detection of emotionally significant stimuli (LeDoux, 2000). Several previous studies found amygdala reactivity to the sound of a crying infant, possibly indicating that the amygdala is involved in the detection of threat cues that signal that the infant is in danger (Riem et al., 2014; Seifritz et al., 2003). Amygdala reactivity has also been found in response to infant laughter (Riem et al., 2012b; Sander, Brechmann, & Scheich, 2003; Seifritz et al., 2003). Thus, the amygdala seems to play a role in the detection of both happy, rewarding infant signals and expressions of infant distress. It is, however, unknown whether the amygdala is also involved in the representation and encoding of infant temperament

In addition to amygdala activity, *connectivity* between the amygdala and other brain regions involved in emotional processing also plays a role in the perception of infant signals (Atzil, Hendler, & Feldman, 2011). The amygdala is suggested to be a functional connectivity hub because of its widespread connections to other brain regions involved in emotional processing (Pessoa, 2010; Pessoa & Adolphs, 2010). For example, the amygdala has strong reciprocal connections to the visual cortex and these connectivities facilitate the perceptual tuning in the visual sensory cortex based on stimulus evaluation and significance. Visual cortex responses to emotionally salient stimuli depend on inputs received from the amygdale, and this process serves the upregulation of processing emotionally significant stimuli (Pessoa, 2008, 2010; Pessoa & Adolphs, 2010; Vuilleumier & Pourtois, 2007), such as positive or negative infant signals. In addition, amygdala connectivity with frontal regions, such as the orbitofrontal cortex and the medial prefrontal cortex, is important for encoding the rewarding value and associative information about the motivational significance of stimuli (Murray, 2007; Schoenbaum, Chiba, & Gallagher, 2000). In a previous study, we found increased amygdala connectivity with frontal reward areas during exposure to infant laughter after administration of the hormone oxytocin (Riem et al., 2012b). Thus, amygdala connectivity seems to be involved in the encoding of the rewarding value of infant signals and may also be involved in the representation of a happy infant temperament.

In the current study, we examine neural activity and connectivity in response to infants with experimentally manipulated temperament using a paradigm called the Baby Social Reward Task (BSRT) (Bhandari et al., 2014; Parsons et al., 2014). The BSRT is a probabilistic learning task where child temperament is derived from learning the facial and vocal features indicative of a more or less happy or sad baby. The BSRT consists of three parts: The first phase is a baseline measure of perceived temperament and cuteness. In addition, participants indicate their motivation to see the infant faces by key pressing to control the length of time the faces are onscreen. The second phase is training where, over a series of trials, participants learn to differentiate more often sad and more often happy infants by observing the infants’ facial expressions and vocalizations. After the training phase, participants evaluate the infants again in terms of temperament, cuteness, and motivation to see the infant. The BSRT has been used to show that the perception of infant temperament and cuteness is not based on physical facial features alone, but is modifiable through experience (Bhandari et al., 2014; Parsons et al., 2014).

Here, we use the BSRT to compare brain activity during the perception of neutral faces of infants with happy or sad temperaments to the activity during the perception of neutral infant faces without temperamental cues. This experimental procedure is a unique window to study variations in brain activity triggered by infants with different temperaments under controlled experimental conditions. We expect that the amygdala is involved in the encoding of infant temperament and shows elevated reactivity to infants with a sad temperament, even in the absence of salient emotional expressions of the infant. In addition, we expect that amygdala connectivity is related to ratings of infant temperament and involved in explaining individual differences in the perception of infants with different temperaments.

## Method

### Participants

Participants were 54 female undergraduate students from the Department of Child and Family Studies, Leiden University. Participants were screened for MRI contraindications, childhood experiences, psychiatric or neurological disorders, hearing problems, pregnancy, alcohol and drug abuse, and did not have children of their own. See Riem et al. (in press) for the recruitment and selection of participants. More than 95% of the participants were born in The Netherlands. Two participants were excluded due to excessive head movement and two participants were excluded because fMRI scanning could not be completed due to technical or health problems during the session. Three participants were excluded from the analysis because they did not learn to discriminate between the sad and happy infants and their mean accuracy scores were two standard deviations below the mean on the final part of the learning phase of the BSRT. This resulted in a total sample size of 47 participants for the current study. The mean age of the participants was 19.62 (SD = 2.12). Written informed consent was obtained from all participants. Permission for this study was obtained from the Institute’s Ethics Committee and from the Leiden University Medical Centre Ethics Committee.

### Procedure

Participants were invited for a lab session at the Leiden University Medical Center. They first signed the consent form and were screened for MRI contraindications. Afterwards, the Baby Social Reward Task (BSRT) was administered. The BSRT was used to manipulate and measure participants’ perception of the temperament of six babies (see Parsons et al., 2014, for a detailed explanation of the paradigm). The task consisted of three different phases: baseline measures of participants’ responses to the infant faces, the experimental manipulation of infant temperament, and post-manipulation measures of participants’ responses to the infant faces.

The first phase consisted of two tasks: the Rating task and the Wanting task (Parsons et al., 2014). In both tasks each baby’s face is presented on the middle of the screen with a neutral expression. In the Rating task the participant is asked to evaluate the babies on three different dimensions (“cuteness,” and two temperament dimensions “difficultness” and “easiness”) pressing the “up” or “down” arrow on the keyboard to change the level of a vertical visual analogue scale (VAS). Similar to Parsons et al. (2014), the ratings of difficultness and easiness were then combined (the difficultness values were reversed) to produce a global measure of temperamental easiness of the baby. Stimuli were presented in random order for 5 s and each participant rated each face once. The Wanting task is a measure of motivation to see each baby’s face. The participants are asked to press the “up” or “down” arrow to change the time they want to see the baby. A vertical VAS with a descending level represented the passing of time and the participant’s key pressing changed the speed of the descending level (pressing the “up” arrow decreased the speed of the descending level, extending the amount of time the face was onscreen, while pressing the “down” arrow increased its speed, reducing the amount of time the face was onscreen). Data regarding the baseline ratings of the baby faces are presented in the Appendix.

The second part of the BSRT consisted of the manipulation of the temperament: in each trial the participants were presented with one of three pairs of baby faces and they were asked to identify, by trial and error, the happy baby or the sad baby of the pair. Participants selected one of the two babies and received feedback, in the form of a change of facial expression and an equivalent vocalization (either “happy” or “sad”). By means of repeated trials, participants could infer how often the baby cried or laughed and decide which one was the happier or the sadder of the two. Similar to Parsons et al. (2014), participants were instructed that “In each pair of faces, there is one happy and one sad baby. Like in real life the happy baby will not always be happy and the sad baby will not always be sad. In each set your task is to find the happier baby, the one who smiles most often, and continue to always select this baby even if this baby may sometimes appear sad.” The three pairs of babies varied in the probability of each infant of being happy or sad. In the easy pair, the easy-to-learn *happy* infant laughed in 80% of trials and cried in the remaining 20% of trials. The easy-to-learn *sad* infant laughed in 20% of trials and cried in the other 80% of trials. In the difficult-to-learn pair the difficult-to-learn *happy* infant laughed 60% of the time while the difficult-to-learn *sad* infant laughed 40% of the time. As a variation to the BSRT task used in previous works (Parsons et al., 2014), we adapted the procedure to the fMRI requirements by eliminating a third pair (with 70% of probability for the “happy baby” to be happy) and instead introduced a “neutral pair” where no feedback was given. Participants were informed about this neutral pair and were told that they would not receive feedback about one pair of infants. See Table 1 for an explanation of the labels that are used for each infant in the current study. Participants received feedback just for the selected baby, but they could infer that the infant not selected would show the opposite emotion on each trial. In each trial, neutral faces were presented onscreen until participants made a response. After participants made a response, visual feedback was presented immediately for 1.5 s accompanied by a 1.5-s vocalization. There was a 500-ms gap between the end of the feedback and the beginning of the next trial during which a red fixation cross was presented in the center of the screen.

**Table 1:** Labels and explanations for the 80%, 60%, 40%, and 20% happy babies and the neutral babies

Label	Explanation	Likelihood of positive expression
Easy to-learn *happy* Baby	The happy baby in the easy pair	80%
Easy-to-learn *sad* Baby	The sad baby in the easy pair	20%
Difficult-to-learn *happy* Baby	The happy baby in the difficult pair	60%
Difficult to-learn *sad* Baby	The sad baby in the difficult pair	40%
Neutral	No cues about temperament	–

The training consisted of two blocks of 60 trials each, so that each pair was presented 40 times in total (20 times per block). In one of the two blocks the participant was asked to select the happy baby, and in the other to select the sad baby. The order of the trials was randomized within session and the order of the blocks was randomized between participants. The identity of the babies (happy, sad, or neutral) was randomized between participants.

After the training phase participants were ready to start the fMRI procedure. Participants were asked to change clothes and were given instructions about the fMRI paradigm (see fMRI paradigm and data acquisition). The third phase of the BSRT, consisting of the post-manipulation evaluation of the babies, was administered after the fMRI paradigm. During this phase participants were asked to perform the Rating and Wanting tasks again. Participants rated the cuteness, easiness, and difficultness of the babies and indicated through button-press how long they wanted to see the babies. See the Appendix for data regarding the post-manipulation ratings of the baby faces. Comparisons of the pre- and post-manipulation ratings and wanting data showed that the manipulation of temperament was effective. The pre-manipulation measurements did not show significant differences in perceived temperament between the infants (see supporting information in the Supplemental Material). The BSRT was programmed and performed using Presentation software (Version 14.4 Neurobehavioral Systems, Inc., www.neurobs.com).

### Stimuli

All infant faces images and vocalizations were the same as those used in Parsons et al. (2014) and Bhandari et al. (2014). The pictures represented smiling, crying, and neutral faces for each of the six babies (aged 3–12 months) of the set. In order to reduce any confounding effects of stimulus gender, we had an independent sample of adult females (n = 40) rate faces from a larger set of 13 (Kringelbach et al., 2008) as “male,” “female,” or “cannot tell.” These ratings were then used to select six faces such that there were two faces clearly perceived as female, two as male, and two with ambiguous ratings (Parsons et al., 2014). Images were in grayscale and equal in size (300×300 pixels) and luminosity. Vocalizations were six laughing babies and six crying babies, as unambiguously evaluated by adults (Young et al., 2012), and sampled from the larger Oxford Vocal (OxVoc) Sounds Database, which is a validated set of non-acted affective sounds from human infants, adults, and domestic animals (Parsons et al. 2014). The vocalisations were 1.5 s long, free from background noise, and matched for the characteristics of the sounds. Vocalizations were presented to the participants through headphones. The infant vocalizations and emotional expressions were only used during the training phase of the BSRT. During the fMRI task, only neutral facial expressions were presented to participants.

### fMRI paradigm and data acquisition

Inside the MRI scanner, participants were presented with the six infant faces *with neutral facial expressions*, one at a time. Each neutral infant face was presented in the center of the screen and accompanied by the words “sad” and “happy.” Participants were asked to indicate if the infant was happy or sad by using button presses with the right hand, based on what they had learned during the training phase of the BSRT. Infant faces were presented 20 times for a maximum of 2.6 s, in random order, resulting in a total of 120 infant presentations. The task was self-paced, meaning that the task continued to the next trial after a button press. Interstimulus intervals were jittered and calculated using Optseq (https://surfer.nmr.mgh.harvard.edu/optseq/). The mean number of errors during the fMRI paradigm was calculated, excluding the trials with the neutral infants because there was no correct answer for these infants. The percentage of incorrect trials was 26.3% (M = 21.04, SD = 17.58). Because of the low number of incorrect trials within each condition, it was not possible to reliably examine brain activation during correct versus incorrect trials during the fMRI task. The fMRI paradigm was programmed and administered using E-Prime software (version 2.0).

### fMRI data acquisition and analysis

Scanning was performed with a standard whole-head coil on a 3-T Philips Achieva TX MRI system (Philips Medical Systems, Best, The Netherlands) in the Leiden University Medical Center. For fMRI, a total of 298 T2*-weighted whole-brain echoplanar images were acquired (repetition time = 2.2 s; echo time = 30 ms, flip angle = 80°, 38 transverse slices, voxel size 2.75 × 2.75 × 2.75 mm (+10% interslice gap)). Following the fMRI scan, a T1-weighted anatomic scan was acquired (flip angle = 8°, 140 slices, voxel size .875 × .875 × 1.2 mm).

The following pre-statistics processing was applied: motion correction (MCFLIRT, Jenkinson et al., 2002), non-brain removal (Smith, 2002), spatial smoothing using a Gaussian kernel of full-width-at-half-maximum 8.0 mm, and highpass temporal filtering (highpass filter cutoff = 90.0 s). Functional scans were registered to the high-resolution EPI-images (high-resolution functional scans), which were registered to the T1-weighted images, which were registered to standard space (Jenkinson et al., 2002).

Data analysis was carried out using FEAT (FMRI Expert Analysis Tool) version 6.00, part of FSL (Smith et al., 2004). In native space, functional activity was examined using general linear model analysis. Each infant (easy-to-learn *happy*, difficult-to-learn *happy*, easy-to-learn *sad*, difficult-to-learn *sad*, neutral 1, neutral 2) was modeled separately as a square-wave function. Each predictor was then convolved with a double gamma hemodynamic response function and its temporal derivative was added to the model, giving six regressors. To examine brain regions involved in the perception of infants with different temperaments, we contrasted the easy-to-learn *happy*, the difficult-to-learn *happy*, the easy-to-learn *sad*, and the difficult-to-learn *sad* infant with one of the neutral infants (easy-to-learn *happy* > neutral, difficult-to-learn *happy* > neutral, easy-to-learn *sad* > neutral, difficult-to-learn sad > neutral).

In addition, we examined psychophysiological interactions (PPI), that is, condition-dependent changes in the covariation of the response between a seed region and other brain regions (Friston et al., 1997). We used the left and right amygdala as seed regions. We extracted the mean time series for each participant from the left and the right amygdala, defined using the Harvard–Oxford subcortical atlas. These time series were then used as a physiological regressor in the model. We applied two separate models: one to analyze left amygdala connectivity, and one to study right amygdala connectivity. Contrasts for the easy-to-learn *sad*, easy-to-learn *happy*, difficult-to-learn *sad*, difficult-to-learn *happy* infant, and the neutral infant (all conditions versus baseline) were created. These regressors were convolved with a double gamma hemodynamic response function and their temporal derivatives were added to the model. The easy-to-learn *sad*, easy-to-learn *happy*, and the neutral infant contrasts were used as psychological regressors. Finally, the interaction between the psychological regressors and the time series from the left or right amygdala were modeled. We assessed the positive and negative contrast of the interaction in order to examine condition-dependent changes in functional connectivity.

All first-level contrast images and the corresponding variance images were transformed to standard space and submitted to second level mixed-effects group whole brain analyses. The group mean was tested using one-sample t-tests on these contrasts and the reverse contrasts (neutral > easy-to-learn *happy*, neutral > difficult-to-learn *happy*, neutral > easy-to-learn *sad*, neutral > difficult-to-learn *sad*). For PPI analysis, we tested the group means using one-sample t-tests on the positive and negative contrasts of the psycho-physiological interaction. We only examined functional connectivity during the easy-to-learn happy and sad infants because behavioral and functional activation results indicated that the easy-to-learn happy and sad infants were more emotionally salient compared to the difficult-to-learn happy and sad infants. We included the number of errors of the last ten trials of the training phase of the BSRT as a confound regressor in the functional activity analysis and PPI analysis. The statistical images were thresholded using clusters determined by *Z* > 2.3 and a cluster corrected significance threshold of *p* < .05. Moreover, because of concerns about Type 1 errors (Eklund, Nichols, & Knuttson, 2016), analyses were repeated with a strict threshold of *Z* > 3.0 and *p* < .05. Results from the analyses with the Z > 3.0 contrast are presented in the Supplemental Material.

A whole brain analysis was conducted to examine brain activity during the perception of infants with different temperaments. In addition, a region of interest (ROI) analysis was conducted to examine functional activity in the bilateral amygdala, anatomically defined using the Harvard–Oxford subcortical atlas (http://fsl.fmrib.ox.ac.uk/fsl/fslwiki/Atlases).

Mean Z-values were calculated (using Featquery) for brain regions that were significantly connected to the amygdala, the bilateral occipital fusiform gyrus and the left middle frontal gyrus (anatomically defined using the Harvard–Oxford cortical atlas), in order to examine the relation between amygdala connectivity and the post manipulation ratings of the infants.

## Results

### Behavioral analyses

We examined the relation between the number of errors that were made in the fMRI paradigm and the temperament ratings of the infants after the manipulation. The number of errors was negatively related to the mean post-manipulation rating of the happy infants (easiness and difficultness rating (reversed) combined) (*r* = −.40, *p* < .01) and the difference between the pre and post-manipulation rating of the happy infants (*r* = −.34, *p* < .05), but not to the pre-manipulation rating (*r* = −.09, *p* = .53). Individuals who made fewer errors during the fMRI paradigm rated the happy infants as more positive afterwards, indicating that individuals were accurate during fMRI when the temperament manipulation was effective. The number of errors was not significantly related to the mean post-manipulation rating of the sad infants (easiness, and difficultness rating (reversed) combined) (*r* = .19, *p* = .20).

### Functional brain activation

**Fig. 1:**
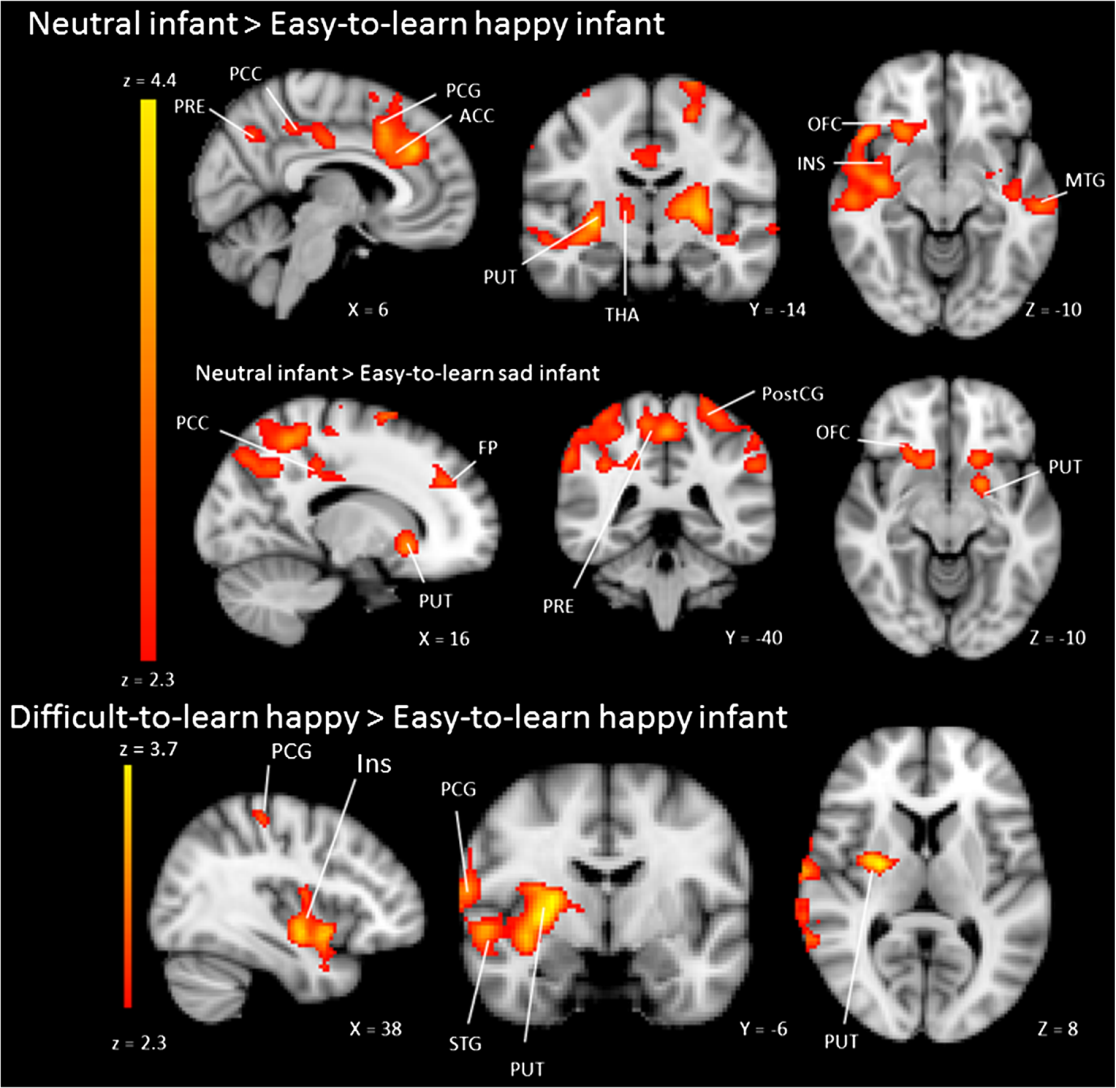
Significant activity during the perception of the neutral infant compared to the easy-to-learn *happy* and *sad* infant and difficult-to-learn *happy* infant compared to the easy-to-learn *happy* infant. Facial expressions of the infants were neutral. *ACC* anterior cingulate cortex, *PCG* paracingulate gyrus, *THA* thalamus, *MTG* middle temporal gyrus, *PostCG* postcentral gyrus, *PRE* precuneus, *PCC* posterior cingulate cortex, *PUT* putamen, *FP* frontal pole, *OFC* orbitofrontal cortex, *INS* insula, *SMG* supramarginal gyrus, *STG* superior temporal gyrus. The right side of the brain corresponds with the left hemisphere and vice versa. Statistical images were thresholded with clusters determined by *Z* > 2.3 and a cluster-corrected significance threshold of *p* < 0.05

**Fig. 2:**
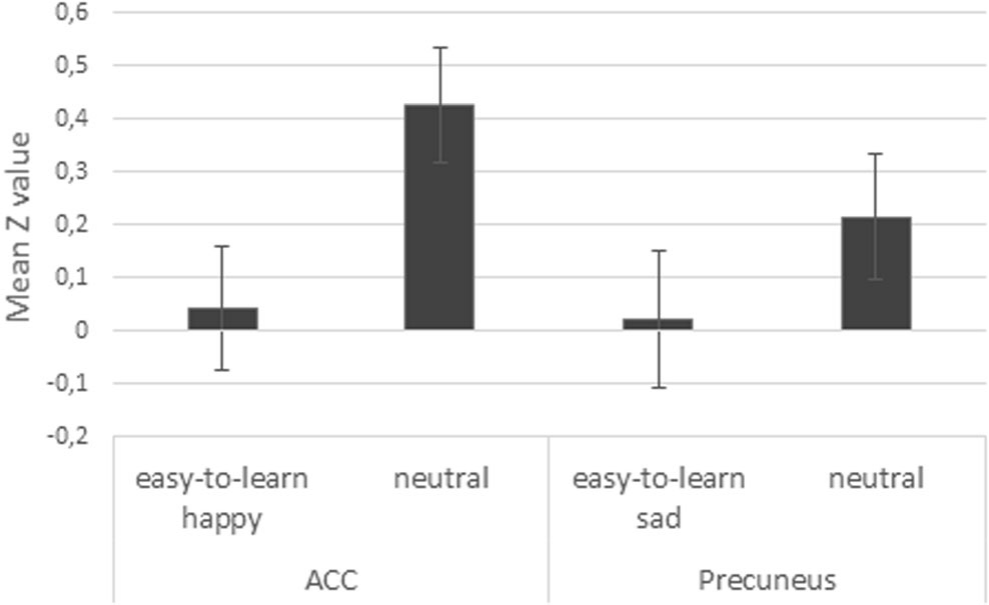
Mean and SE Z values of anterior cingulate cortex activity during presentation of the easy-to-learn *happy* infant and neutral infant and precuneus activity during the easy-to-learn *sad* infant and neutral infant

A whole brain analysis was conducted to examine brain activity during the perception of neutral infant faces associated with a happy, sad, or neutral temperament. We assessed the contrasts (i) easy-to-learn *happy* infant versus neutral infant, (ii) difficult-to-learn *happy* infant versus neutral infant, (iv) easy-to-learn *sad* infant versus neutral infant (v) difficult-to-learn *sad* infant versus neutral infant, and the reverse contrasts. The contrast difficult-to-learn *happy* infant versus neutral infant showed significant activity in the cuneal cortex, but no significant activity was found during the perception of the easy-to-learn *happy* infant, the easy-to-learn *sad* infant, the difficult-to-learn *happy* infant, and the difficult-to-learn *sad* infant compared to neutral infants. However, the reverse contrasts, comparing activity during neutral infants with easy-to-learn *sad* or easy-to-learn *happy* infants, revealed significant activity in several brain regions (see Table 2 and Figs. 1 and 2). We found significant activity during the perception of the neutral infant compared to the easy-to-learn *happy* infant in the middle frontal gyrus, orbitofrontal cortex, the frontal pole, the angular gyrus, the putamen, the anterior cingulate cortex, precuneus, the middle and superior temporal gyrus, the insula and the paracingulate gyrus. In addition, during the perception of the neutral infant compared to the easy-to-learn *sad* infant, significantly more activity was found in the postcentral gyrus, the precuneus, the frontal pole, the orbitofrontal cortex, the anterior cingulate cortex, the paracingulate gyrus, the thalamus, the nucleus accumbens, and the putamen (see Table 2 and Figs. 1 and 2). The ROI analysis with the amygdala showed significant amygdala activity during the perception of the easy-to-learn *happy* infant compared to the neutral infant. No significant amygdala activity was found during the perception of the easy-to-learn or difficult-to-learn *sad* infant or difficult-to-learn *happy* infant compared to the neutral infant.

**Table 2:** MNI coordinates, cluster size, and Z-max values for significantly activated clusters revealed by the whole brain analysis and ROI analysis with the amygdala ^a^

Contrast	Brain region	N voxels	Z max	MNI coordinates Z max		
				x	y	z
Neutral > Easy-to-learn *Happy*	L middle frontal gyrus	7,305	4.20	−32	24	28
	R angular gyrus	7,296	4.50	54	−48	40
	R putamen	3,381	4.08	32	−16	0
	L putamen	1,533	4.01	−26	−14	4
	R amygdala^a^	14	2.61	18	−14	−12
Difficult-to-learn *Happy* > Neutral	R cuneal cortex	2,165	3.50	8	−80	34
Difficult-to-learn *Happy* > Easy-to-learn *Happy*	R putamen	4,355	3.74	26	−6	8
Neutral > Easy-to-learn *Sad*	R postcentral gyrus	8,787	3.87	52	−28	56
	R frontal pole	1,980	4.00	26	46	24
	R nucleus accumbens	1,189	3.58	14	18	−6

In additional analyses, we assessed the contrasts easy-to-learn *happy* infant versus difficult-to-learn *happy* infant and easy-to-learn *sad* infant versus difficult-to-learn *sad* infant. There was significantly more activity in the supramarginal gyrus, postcentral gyrus, middle and superior temporal gyrus, insula, and putamen during the perception of the difficult-to-learn *happy* infant compared to the easy-to-learn *happy* infant (see Fig. 1). The pattern of activation partially overlapped with brain activity resulting from the neutral infant compared to the easy-to-learn *happy* infant. The contrast easy-to-learn *sad* infant versus difficult-to-learn *sad* infant did not reveal significant activity.

### Functional connectivity

**Fig. 3:**
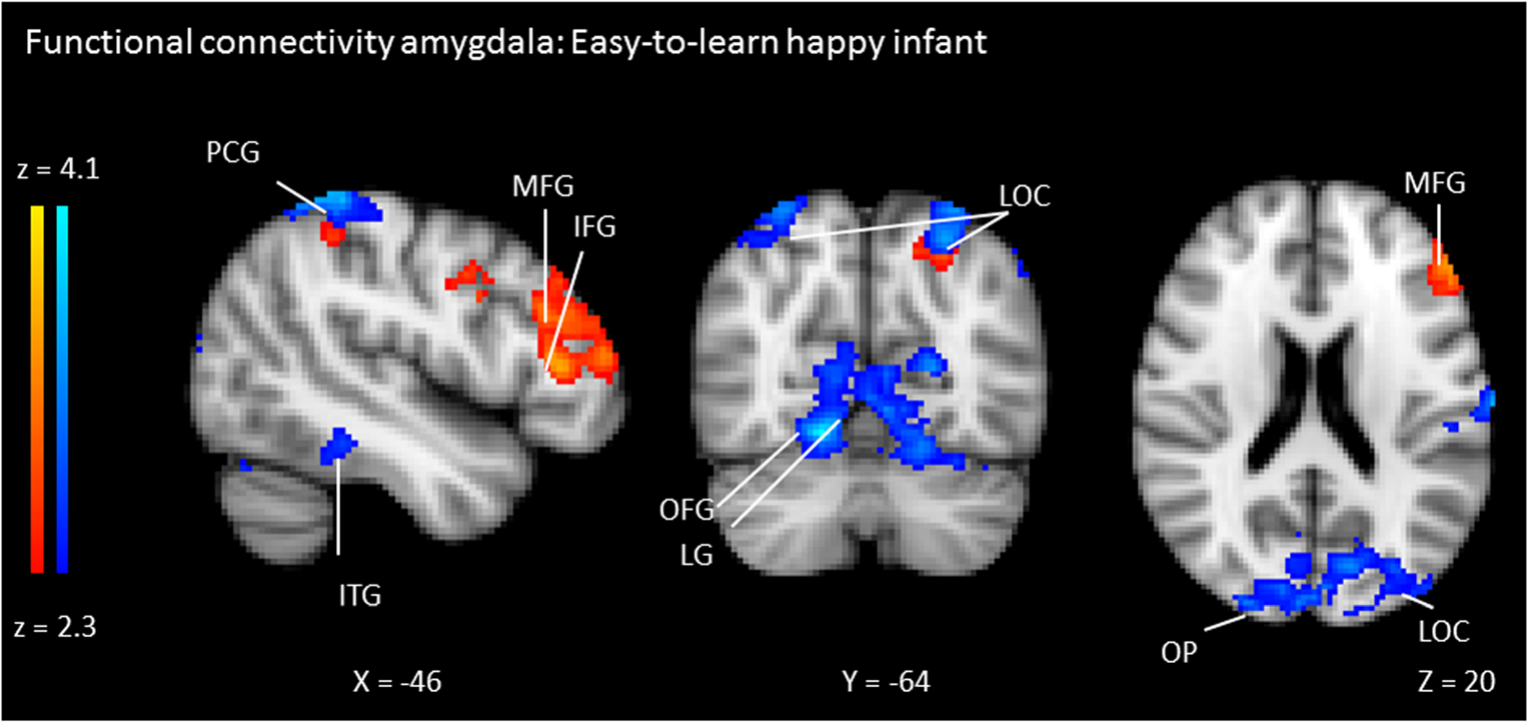
Significant functional connectivity with the left (red) and right (blue) amygdala during the presentation of the easy-to-learn *happy* infant (with a neutral facial expression). *ITG* inferior temporal gyrus, *PCG* postcentral gyrus, *MFG* middle frontal gyrus, *IFG* inferior frontal gyrus, *LG* lingual gyrus, *LOC* lateral occipital cortex, *OFG* occipital fusiform gyrus, *OP* occipital pole. The right side of the brain corresponds with the left hemisphere and vice versa. Statistical images were thresholded with clusters determined by *Z* > 2.3 and a cluster-corrected significance threshold of *p* < 0.05

We performed PPI analyses to examine amygdala connectivity during the perception of infants with different temperaments. When participants were presented with the easy-to-learn *happy* infant (vs. baseline), there was significant connectivity between the right amygdala and the bilateral occipital fusiform gyrus (OFG), the lateral occipital cortex, the occipital pole, the postcentral gyrus, inferior temporal gyrus, and the lingual gyrus and between the left amygdala and the left middle frontal gyrus (MFG), the frontal pole, the inferior frontal gyrus, the postcentral gyrus, and the lateral occipital cortex (see Table 3 and Fig. 3 for the clusters of connectivity and Table S1 for the local maxima in the clusters). In addition, we found significant functional connectivity between the left amygdala and the left superior frontal gyrus during the presentation of the neutral infant (vs. baseline). There was no significant amygdala connectivity during the presentation of the easy-to-learn *sad* infant.

**Table 3:** Overview of functional amygdala connectivity: MNI coordinates, cluster size, and Z-max values for significant clusters of functional connectivity

Contrast	Direction	Seed region	Functional connectivity	N voxels	Z max	MNI coordinates Z max		
						x	y	z
Easy-to-learn *Happy*	-	Right amygdala	Lingual gyrus	8,363	4.12	16	−64	−12
	-	Left amygdala	Middle frontal gyrus	1,092	3.68	−52	32	24
			Lateral occipital cortex	927	3.32	−26	−62	50
Neutral	+	Left amygdala	Superior frontal gyrus	1,089	3.32	−8	0	72

Furthermore, mean Z-values were extracted from the OFG and the MFG in order to examine the relation between amygdala-OFG/MFG connectivity and the post-manipulation ratings of the infants. There was a significant positive correlation between amygdala-OFG connectivity and the post-manipulation temperament rating of the happy infants (*r* = .31, *p* = .03, not significant after Bonferroni correction) (see Fig. S4 for a scatterplot). Individuals with high levels of amygdala-OFG connectivity during the presentation of the easy-to-learn *happy* infant rated the happy infants as more positive than individuals showing low levels of functional connectivity. A contrast Z-value was calculated by subtracting the mean Z-value for the OFG during the presentation of the neutral infant from the mean Z-value for the OFG during the presentation of the easy-to-learn *happy* infant. The correlation between the contrast Z-value (easy-to-learn *happy* infant vs. neutral infant) and the post-manipulation temperament rating of the happy infants was significant (*r* = .30, *p* = .04). There was no significant correlation between amygdala-OFG connectivity and the post-manipulation cuteness ratings of the happy infants (*r* = −.06, *p* = .71) or time wanting to view the happy infants (*r* = −.04, *p* = .81). Neither were there significant correlations between amygdala-MFG connectivity and the post-manipulation ratings of the happy infant (all *p* > .17).

## Discussion

The current fMRI study is the first to experimentally manipulate infant temperament and examine subsequent neural processing of infant faces. We employed a computerized probabilistic learning task that manipulated infant temperamental features, the BSRT, to create a sense of the temperament of previously unfamiliar infants and examined its effects on neural processing. Our study is the first to demonstrate how a simple temperament manipulation can change brain activity to a basic social reward, namely infants with a neutral facial expression. We found that exposure to infants with a happy or sad temperament resulted in decreased activation in a neural network involved in emotion processing compared to infants without temperamental cues. In addition, our results indicate that amygdala connectivity is involved in explaining individual differences in the perception of infant temperament, which is in line with previous studies indicating that the amygdala plays an important role in the perception of infant signals (Barrett et al., 2012; Kim et al., 2011; Riem, Bakermans-Kranenburg, van Ijzendoorn, Out, & Rombouts, 2012a; Riem et al., 2011). Our study indicates that previous positive or negative experiences with an infant influence how the brain responds to that infant. Infants with a sad or happy temperament elicit different brain activity and connectivity than infants with an unknown temperament, even when the facial expression is neutral and the same for all infants. This effect cannot be accounted for by a familiarity effect, for all infant faces were presented with the same frequencies.

Infant faces without temperamental cues resulted in increased activation in a neural network involved in emotional processing compared to infant faces with a happy or sad temperament. More specifically, neutral infants elicited activation in brain regions involved in empathy, including the insula and anterior cingulate cortex (Lamm, Decety, & Singer, 2011), and brain regions involved in theory of mind and mentalizing, including the precuneus and middle and superior temporal gyrus (Carrington & Bailey, 2009; Van Overwalle & Baetens, 2009). One explanation for this finding is that these neutral infant faces may have been perceived as ambiguous. Although participants had seen the neutral infants as often as the infants with the happy and sad temperament, they did not “know” the infants in terms of their temperament and emotionality. Categorizing infants without temperamental cues as being happy or sad is more difficult, requires more effort, and may therefore result in more neural activation. Knowing the temperament of an infant seems to be adaptive because it requires fewer neural resources, regardless of whether the infant has a happy or sad temperament. Thus, information about infant temperament may facilitate the interpretation of infant signals and the selection of an appropriate caregiving response.

Contrary to our expectations, we did not find increased amygdala activity in response to infants with a sad temperament. This seems to be in contrast to previous studies pointing towards a role in the processing and perception of infant distress (Riem et al., 2011). One explanation for the absence of amygdala activity is that we contrasted amygdala activity during the perception of sad and happy infants with neutral infants. The amygdala also responds to neutral faces (Pessoa, 2010), which might explain why the sad versus neutral contrast did not reveal significant amygdala activity. In contrast to previous studies that found amygdala responses to sad infant faces or infant cry sounds, we did not present participants with stimuli with clear threat signals: the facial expression of the infants were neutral in all conditions. The amygdala shows a rapid and automatic response to threat, independent of context, attention, and awareness (Dolan & Vuilleumier, 2003). A sad infant temperament is not a direct alarming signal of threat and may therefore not elicit different amygdala activity compared to neutral infant faces.

Interestingly, we found increased amygdala connectivity during the perception of infants with a happy temperament. This is consistent with the suggestion that amygdala connectivity is involved in encoding the rewarding value of stimuli (Murray, 2007; Schoenbaum et al., 2000). Moreover, amygdala connectivity during the perception of the happy infants was related to the post-manipulation temperament rating of the happy infants. Individuals with high levels of amygdala-OFG connectivity rated the happy infants as more positive than individuals showing low levels of functional connectivity. The amygdala is functionally and anatomically connected to the regions of the visual cortex, including the fusiform gyrus, and exerts a modulatory influence on visual cortex responses based on the biological and affective relevance of the stimulus. In this way, the amygdala prioritizes the processing of emotional stimuli over others and separates significant from less significant stimuli (Pessoa, 2010). The fusiform gyrus is particularly important for face processing and receives amygdala projections that serve enhanced processing of emotional faces (Herrington, Taylor, Grupe, Curby, & Schultz, 2011; Vuilleumier, Richardson, Armony, Driver, & Dolan, 2004). Our finding that amygdala-occipital fusiform gyrus connectivity is related to the temperament ratings of the happy infants might therefore indicate that the amygdala plays an important role in enhancing the processing of positive emotional infant stimuli and tagging infant stimuli such as infant laughter as emotionally significant, in particular in nulliparous females. Surprisingly, amygdala connectivity during the perception of the sad infant was not related to temperament ratings of the sad infant, possibly because the participants perceived the sad infant as less emotionally salient. Indeed, a comparison of the pre- and post-manipulation ratings of temperament indicated that only the happy infants were perceived as more positive and cute after the training phase of the BSRT. In line with our findings, a previous study indicated that non-parents are particularly sensitive to infant laughter and show less amygdala reactivity to crying than to laughter (Seifritz et al., 2003). In contrast, parents showed more amygdala reactivity to crying than to laughter. Thus, this vocalization-specific pattern of response seems to change after the transition to parenthood, which may be important for the adaptation to the specific demands associated with successful infant care (Seifritz et al., 2003).

Our study has a few limitations. First, functional connectivity using fMRI is a correlational method that does not allow conclusions about (the direction of) any causal relation between the amygdala and other brain regions. The development of new whole-brain computational modelling methods may in the future allow for further investigations of the underlying causal mechanisms (Deco & Kringelbach, 2014; Deco et al. 2015). Second, our findings can only be generalized to women without children of their own. Women without children were recruited for participation in order to control for influences of parenting experiences with own infants. The results may be different in parents because amygdala reactivity to infant signals are influenced by parental status (Seifritz et al., 2003). Since infant crying can trigger harsh caregiving responses and even child abuse and neglect (Barr, Trent, & Cross, 2006; Reijneveld, Van der Wal, Brugman, Sing, & Verloove-Vanhorick, 2004), it is important to examine how infants who cry more often than others are perceived by their parents and how that relates to caregiving behavior. It should be noted that temperament is a complex construct that was simplified to only one dimension ranging from happy to sad in the current study. Other aspects of infant temperament such as activity and inhibition are more difficult to artificially model in an experimental paradigm like the BSRT. However, experimental manipulation is not possible in natural contexts because it would require rather forceful interventions. The advantage of our simplified, one-dimensional manipulation of temperament, however, is the more unequivocal, focused interpretation of a very salient temperamental characteristic. Finally, an untrained neutral condition was used as a baseline condition in this study. Enhanced brain activity during an untrained neutral condition may be related to the lack of training, e.g. not being able to draw on memory or not knowing the right response. However, this is unlikely since we also found enhanced activity during the perception of the difficult-to-learn *happy* infant compared to the easy-to-learn *happy* infant, even though participants were reliably trained to identify both infants as happy and rated both infants as cuter and more positive after the training phase (see Supplemental Material). This indicates that even subtle differences in infant temperament are associated with changes in brain activity. Thus, although the difficult-to-learn *happy* infant and the easy-to-learn *happy* infant were both perceived as happy infants, they elicited differential patterns of brain activity, indicating that previous experiences with an infant influence brain responses to that infant.

In sum, we examined how computerized manipulation of infant temperamental features affects subsequent neural processing of infant faces as assessed with fMRI. To our knowledge, this is the first demonstration of how temperament manipulation can change brain responses to infant signals. We found that information about infant temperament results in decreased activation of a neural network involved in emotion processing, possibly indicating that infant temperament is a source of contextual information that facilitates the interpretation of infant signals. Our findings point to a role of the amygdala in the perception of infant signals and indicate that amygdala connectivity is involved in the representation of infant temperament. Amygdala connectivity appears to be important for the encoding of the rewarding value of an infant with a happy temperament, which may affect subsequent caregiving responses.
